# The different effects of aspect ratios of letters on the letter-row tilt illusion in staircase and non-staircase stimuli

**DOI:** 10.3758/s13414-025-03204-5

**Published:** 2026-02-23

**Authors:** Yukyu Araragi, Hiroyuki Ito

**Affiliations:** 1 Institute of Human Sciences, Shimane University, Matsue, Japan; 2 Faculty of Design, Kyushu University, Fukuoka, Japan

**Keywords:** Letter-row tilt illusion, Aspect ratio, Staircase structure, Staircase hypothesis, Horizontal line segments, Non-staircase stimuli

## Abstract

The letter-row tilt illusion is the illusion that the row is perceived to be tilted, when a set of letters is repeated in a physically horizontal or vertical row. We quantitatively examined the effects of aspect ratios of letters on the letter-row tilt illusion in horizontal letter-rows with or without a staircase structure of horizontal line segments. In Experiment 1, the results quantitatively showed that the illusion significantly occurred in letter-rows with and without the staircase structure. In Experiment 2, the results showed that the amount of illusion in letter-rows with the staircase structure increased as relative and absolute lengths of the horizontal line segments increased. In Experiment 3, the results showed that the amount of illusion in letter-rows without the staircase structure had different tendencies in aspect ratios from that with the staircase structure. The present study suggested that different mechanisms were responsible for the letter-row tilt illusions with and without the staircase structure.

## Introduction

**Fig. 1:**
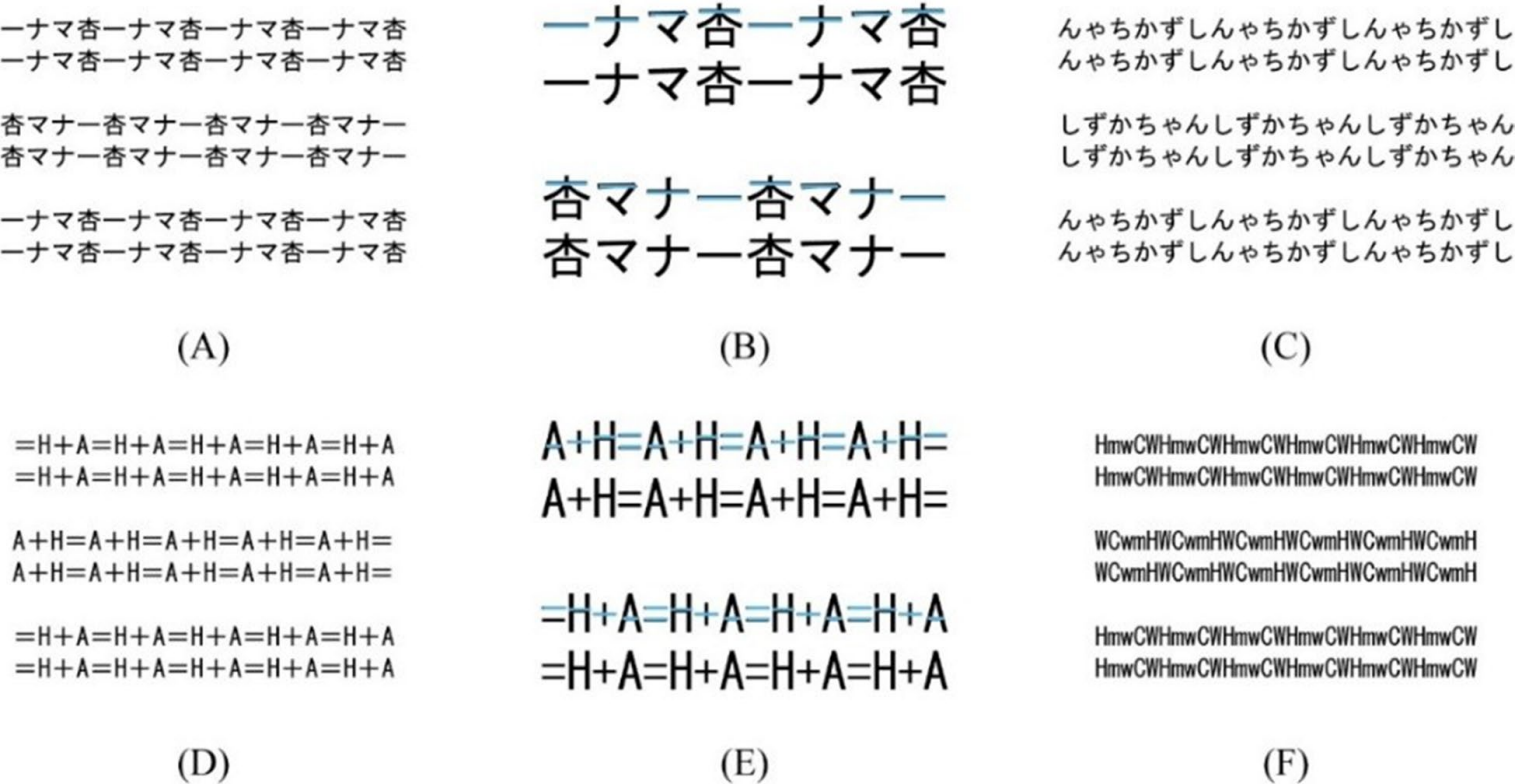
Examples of the letter-row tilt illusion. The letter-rows are in the physically horizontal orientation. The font of all letters in Fig. 1 is MS Gothic. (**A, D**) Letter-rows with a staircase structure of horizontal line segments. In (**A**) and (**D**), the central two letter-rows of six letter-rows are perceived to be tilted clockwise and counterclockwise, respectively. The sets of four letters in the upper/lower and central two letter-rows are repeated leftward and rightward, respectively. (**B, E**) Horizontal line segments represent the staircase structure. Light blue horizontal line segments sequentially step up and down rightward in the upper and lower two letter-rows, respectively. (**C, F**) Typical letter-rows without the staircase structure. The central two letter-rows are perceived to be tilted clockwise

In human vision, many orientation illusions have been reported. The Popple illusion, Zöllner illusion, Café Wall illusion, and twisted cord illusion (i.e., Fraser illusion) are representative examples. Simple geometric structures can generate tilt illusions. A tilt illusion also occurs in letter-rows, although the letter-rows that we see every day do not appear tilted as shown in Fig. 1.

The letter-row tilt illusion first appeared on a Japanese Internet bulletin board system (BBS) in 2005 (Kitaoka, 2007). When a set of Japanese and/or Chinese characters is repeated in a row, the row is perceived to be tilted (hereafter, “characters” are described as “letters” as well as alphabetical letters in the present study). Anonymous BBS users have demonstrated that many letter sets generated the illusion. Figure 1A shows one of the most famous examples (pronounced *anzu manner*; see also Kohara, 2006). The letter sets consist of one Chinese and three Japanese katakana letters in MS Gothic font and do not have a meaning as a word. The rows in the upper/lower and central two letter-rows are perceived to be tilted counterclockwise and clockwise, respectively, although all rows are physically parallel and in the horizontal orientation. The illusion was named the letter-row tilt illusion by Arai and Arai (2005a).

One hypothesis for the letter-row tilt illusion was that the horizontal letter-row is perceived to be tilted counterclockwise and clockwise, respectively, when the horizontal line segment in each letter sequentially steps up and down in a set of letters, as shown in Fig. 1 (Kohara, 2006). We call the hypothesis the staircase hypothesis of horizontal line segments. Kohara (2006) examined the percentage that the letter-rows were perceived to be tilted from the physical horizontal orientation. The stimuli were letter-rows with and without the staircase structure (hereafter, the letter-rows with and without the staircase structure are described as “staircase stimuli” and “non-staircase stimuli,” respectively; in addition, staircase stimuli are defined as letter-rows of letter sets with sequential stairs between all adjacent letters). The letter-rows consisted of letter sets of four Japanese katakana letters. The results showed that the percentage in the letter-rows consisting of letters with horizontal line segments over 9 pixels long was significantly higher than that over 3 pixels long and that of random letters. The percentage over 3 pixels long was significantly higher than that of random letters. Kohara (2006) also examined the perceived angle using the method of adjustment. The results showed that the perceived tilt angle in the letter-rows with horizontal line segments over 9 pixels long was significantly larger than that over 3 pixels long. The results supported the staircase hypothesis, and suggested that the absolute length of horizontal line segments affected the amount of illusion. Arai and Arai (2018) described that sizes of letters affected the illusion, but it was not quantitatively examined. Arai and Arai (2005a) applied the maximal overlap wavelet transform to letter-rows of only one letter set in staircase stimuli, and divided them into stimuli that extracted part of the horizontal components and stimuli that combined the other (i.e. horizontal, vertical, and diagonal) components. Arai and Arai (2005a) reported that the illusion occurred in the stimuli with part of the horizontal components, but the illusion did not occur in the stimulus that combined the other components. (On the other hand, the results in non-staircase stimuli have not been published.) Arai and Arai (2012) applied for a patent for “the letter-row tilt illusion generating device, letter-row tilt illusion generating method, print medium manufacturing method, electronic medium manufacturing method, and program.”

Arai and Arai (2014, 2015) also demonstrated that the illusion occurred in non-staircase stimuli (e.g., “HmwCW” and “PKNcj” in MS Gothic font), as shown in Fig. 1. The demonstration suggested factors for the illusion other than the staircase structure of horizontal line segments. The generation of the illusion in non-staircase stimuli, the reason for the illusion in non-staircase stimuli, or the difference of the mechanisms in staircase and non-staircase stimuli was not quantitatively examined.

The mechanism underlying the illusion remains unclear. Examination of the effect of aspect ratios of letters on the illusion would show the nature of the illusion in the staircase and non-staircase stimuli. We hypothesized that if the underlying mechanism differed in the illusion between staircase and non-staircase stimuli, then the effects of aspect ratios of letters would have different tendencies in staircase and non-staircase stimuli.

The purpose of the present study was to quantitatively examine the effects of aspect ratios of letters on the perceived angle of letter-rows in staircase and non-staircase stimuli. The aspect ratio was defined as the ratio of the horizontal length (width) to the vertical length (height) of each letter. The present study compared the letter sets in the experimental and control conditions in order to examine the effect of aspect ratios of letters on the illusion between the staircase and non-staircase stimuli and to cancel some experimental errors if any. Letters of the letter sets in the experimental and control conditions were the same, but the order of the letters was different. The method of adjustment was used. Experiment 1 quantitatively examined the generation and amount of the illusion in staircase and non-staircase stimuli. Experiments 2 and 3 quantitatively examined the effects of aspect ratios on the perceived angle of letter-rows in staircase and non-staircase stimuli, respectively.

## Experiment 1

### Methods

#### Observers

Ten Japanese undergraduate and graduate students (19–25 years old, *M* = 21.4, *SD* = 2.0; six female, four male) participated as observers. They had normal or corrected-to-normal visual acuity and were not aware of the purpose of the experiment. The experiment was performed with the written informed consent of each observer.

#### Apparatus

The stimuli were presented on a 22-in. CRT monitor (RDF223H, Mitsubishi). A computer (Vostro 3667, Dell) was used to control the presentation of the stimuli and to record responses that the observers made by pressing the assigned key. The observers’ heads were held in position with a forehead and chin rest.

#### Stimuli

**Fig. 2:**
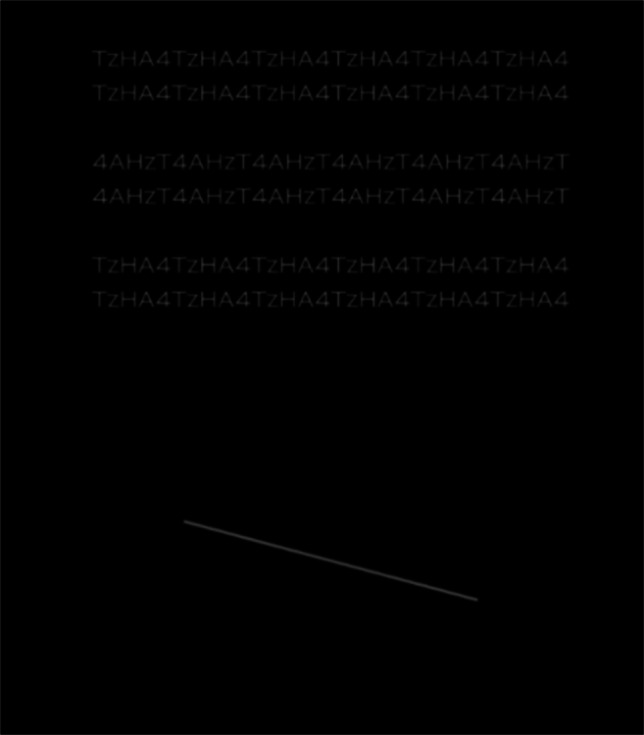
Stimuli in Experiment 1. The stimuli were the letter-rows in the letter set “4AhzT,” experimental, left, upper letter-rows, and clockwise comparison stimuli condition

The stimuli were letter-rows (standard stimulus) and a line segment (comparison stimulus), as shown in Fig. 2. They were presented in the upper or lower parts on the display. Six letter-rows were presented.

Twelve letter sets were used as stimuli with or without meaning as a word as shown in Table 1. The letter-rows of six letter sets (A–F) were the staircase stimuli. The letter-rows of the other six letter sets (G–L) were the non-staircase stimuli. The font of Japanese and Chinese letters was MS Gothic. The font of alphabetic and numeric letters was Copperplate Gothic Light. Letter-rows in the left and right conditions consisted of letter sets (e.g., WCwmH) that were repeated rightward (e.g., WCwmHWCwmHWCwmH) and leftward (e.g., HmwCWHmwCWHmwCW) in the central two letter-rows, respectively. The letter set in the upper and lower two letter-rows was repeated in the opposite direction to that in the central two letter-rows. The letter sets used as stimuli were selected from the previous study, collected from the Internet, or made by the authors in the present study. The letter set A used in the experimental condition was demonstrated in Arai and Arai (2005b), whereas the letter sets C–F were created by the authors in the present study based on the staircase stimuli. The letter sets B, G–K used in the experimental condition were demonstrated on webpages, whereas the letter set L was found by the authors in the present study. The letters used in the experimental and control conditions of each letter set were the same, but the order of the letters was different.

**Table 1:**
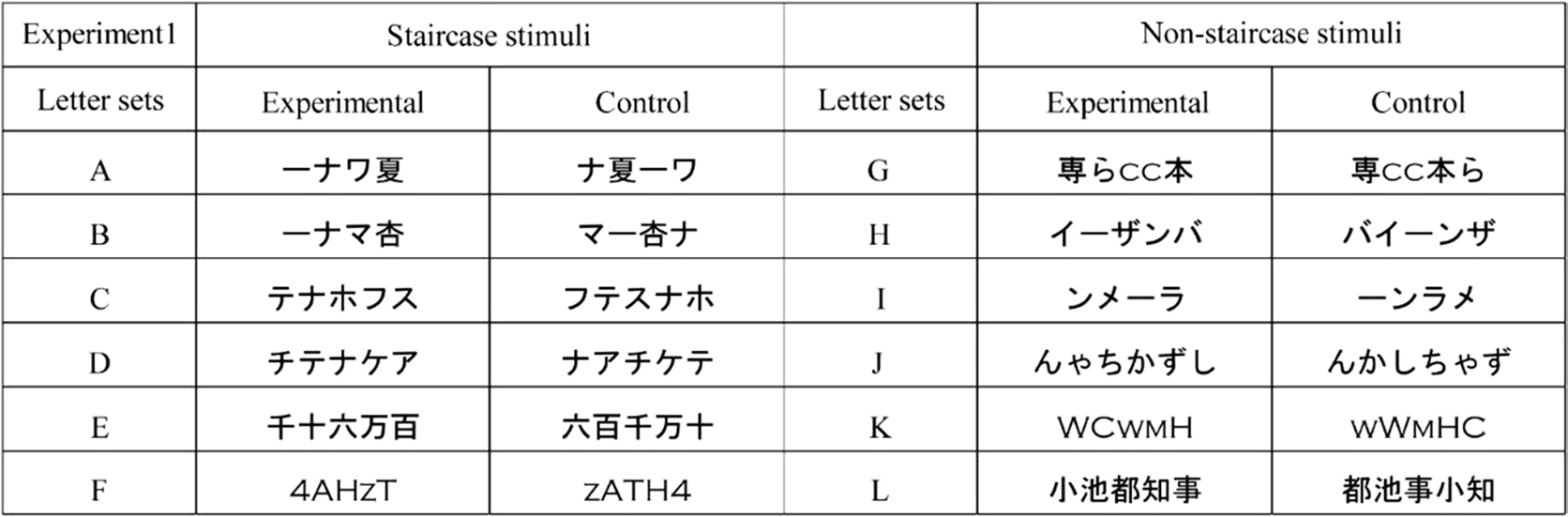
Stimuli of the 12 letter sets used in Experiment 1. The letter sets A, J, and K in the experimental condition were demonstrated in Arai and Arai (2005b, 2014, 2015), respectively. The letter sets G, H, and I, respectively, were shown on each webpage (Science Museum of Visual Illusions, 2018; Zena, 2020; Ponponchiki@ryuseiyarou, 2019)

The horizontally and vertically averaged lengths of two letter-rows over six letter sets were 14.3 and 1.8 degrees, respectively. The stimuli were presented in a square background with a 39.4-degree horizontal and 30.0-degree vertical display. The luminance of the letters and line segment was 26.8 cd/m^2^. The luminance of the black background was 0.01 cd/m^2^. The stimuli were presented until the observers responded by pressing the assigned key.

#### Procedure

The observers viewed the stimuli in a darkened room using binocular vision. The viewing distance was 57.3 cm.

The method of adjustment was used. The observers were requested to rotate the line segment (i.e., comparison stimulus) so that it perceived the angle to be equal to the perceived angle of the central two of six letter-rows (i.e., standard stimulus). The line segment rotated 1 degree clockwise and counterclockwise, respectively, by pressing the “4” and “6” keys on the numerical keypad. The initial orientation of the line segment in a trial was 14 degrees to clockwise or counterclockwise orientation from the horizontal orientation.

Each observer completed two experimental sessions of 96 trials (6 letter sets × 2 standard stimuli × 8 repetitions). The standard stimulus was the experimental or control condition. In each session, the six letter sets were three staircase stimuli and three non-staircase stimuli. Before each session, each observer performed eight practice trials to get accustomed to the task. The order of the trials and the six letter sets in each session were randomized for each observer. The left or right condition, upper or lower letter-rows location, and initial clockwise or counterclockwise orientation of the comparison stimuli were counterbalanced.

### Results and discussion

**Fig. 3:**
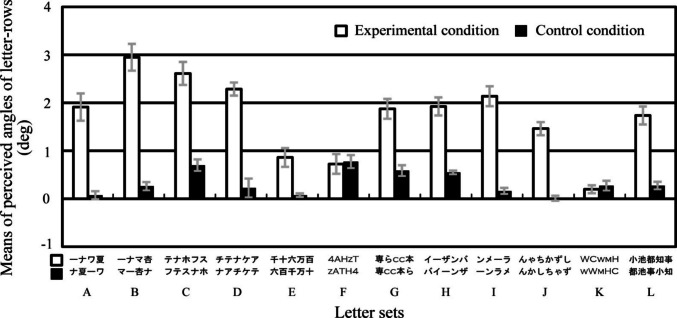
Results of Experiment 1. Means of the perceived angle of the central letter-rows averaged over the ten observers are plotted as a function of the letter sets separately for each standard stimulus. Error bars represent standard errors

Figure 3 shows the means of the perceived angle of the two central letter-rows of the 12 letter sets in Experiment 1. A two-way repeated-measures ANOVA was performed on the perceived angle of the two central letter-rows with standard stimulus (two conditions) and letter set (12 sets) as main factors. The main effect of standard stimulus was significant [*F*(1, 9) = 133.245, *p* <.001, *η*_p_^2^ =.94]. The main effect of letter set was significant [*F*(11, 99) = 22.028, *p* <.001, *η*_p_^2^ =.71]. The interaction was significant [*F*(11, 99) = 15.948, *p* <.001, *η*_p_^2^ =.64]. In ten letter sets, the simple main effect test of standard stimulus showed that the perceived angle was significantly larger in the experimental condition than that in the control condition (letter set A: *F*(1*,* 108) = 43.017, *p* <.001; letter set B: *F*(1*,* 108) = 70.215, *p* <.001; letter set C: *F*(1*,* 108) = 40.199, *p* <.001; letter set D: *F*(1*,* 108) = 107.232, *p* <.001*,* letter set E: *F*(1*,* 108) = 18.791, *p* <.002; letter set G: *F*(1*,* 108) = 45.017, *p* <.001; letter set H: *F*(1*,* 108) = 49.500, *p* <.001; letter set I: *F*(1*,* 108) = 69.862, *p* <.001; letter set J: *F*(1*,* 108*)* = 123.073, *p* <.001; letter set L: *F*(1*,* 108) = 39.987, *p* <.001). The results show that the letter-row tilt illusion significantly occurred in the letter-rows of the ten letter sets. In the other two letter sets, the simple main effect test of standard stimulus was not significant (letter set F: *F*(1*,* 108) = 0.029, *p =*.87; letter set K: *F*(1*,* 108) = 0.343, *p =*.57).

A t-test was performed on the amount of illusion with type of letter set (two types) to compare the amount of illusion between the staircase and non-staircase stimuli. The amount of illusion was calculated as the subtraction of the perceived angle of the letter-rows in the control condition from that in the experimental condition. The statistical units were the means averaged over six letter sets separately for each type and each observer. The results showed that the amount of illusion in the staircase stimuli was significantly larger than that in the non-staircase stimuli (*t*(9) = 2.534, *p =*.03, Cohen’s *d* = 0.801).

These results showed four main points: (1) The staircase stimuli generated the illusion. The perceived angle was significantly larger in the experimental condition than that in the control condition in the letter sets A, B, C, D, and E. (2) The illusion also occurred in the non-staircase stimuli. The perceived angle was significantly larger in the experimental condition than that in the control condition in the letter sets G, H, I, J, and L. The results suggest the existence of the reason for the illusion other than sequential stair structures (i.e., staircase structures) of horizontal line segments. (3) The illusion was attributed to the order of letters (i.e., global structure of the letters) rather than the aggregate of the effect of each letter, since the only difference in the stimuli between the experimental and control conditions was the order of the letters. (4) The amount of illusion in the staircase stimuli was significantly larger than that in the non-staircase stimuli in the letter sets used in Experiment 1.

On the other hand, the results showed that the letter-rows in the two alphabetic letter sets (“4AHzT” (letter set F) and “WCwmH” (letter set K)) did not generate the illusion, since there were no significant differences in the perceived angles between the experimental and control conditions. In the letter set “4AhzT,” there were two possible reasons for the absence of significant differences between the experimental and control conditions. One reason was that letter-rows “4HTAz4HTAz4HTAz” in the control condition unintentionally had four sequential stairs in “z4HT,” and the four sequential stairs might generate the illusion in the control condition. The other reason was that lengths of the horizontal line segments in alphabetic letters seemed to be shorter than those in katakana or Chinese letters. The length of horizontal line segments might not be long enough to generate the illusion. Kohara (2006) showed that the perceived angle in the letter-rows of letters with longer horizontal line segments was significantly larger than that with shorter horizontal line segments, when the horizontal line segments were the sequential stair condition. In addition, Arai and Arai (2018) described that sizes of letters affected the illusion. In the letter set “WCwmH,” the results might be attributed to the difference in the fonts. The font of the stimuli in Experiment 1 was Copperplate Gothic Light, as shown in Table 1. On the other hand, the font of the letters in Arai and Arai (2015) and in Fig. 1 was MS Gothic.

## Experiment 2

Experiment 2 examined the effects of aspect ratios of letters on the perceived angle of letter-rows in the staircase stimuli in order to investigate the generation of the illusion in letter sets of alphabetic and numeric letters and to compare the natures of the illusion in staircase stimuli (Experiment 2) and non-staircase stimuli (Experiment 3). The aspect ratio was defined as the ratio of the horizontal length (width) to the vertical length (height) of each letter (i.e., H-V expansion ratio). We hypothesized that if the horizontal length and vertical length of each letter was long enough, then the illusion would also occur in the letter sets of alphabetic and numeric letters.

### Methods

The methods used in Experiment 2 were identical to those used in Experiment 1, except for the following points. Ten Japanese undergraduate students (19–22 years old, *M* = 20.6, *SD* =1.0; ten female) participated as observers. The six letter sets used in Experiment 2 were the staircase stimuli as shown in Table 2. The font in letter sets A and B was Copperplate Gothic Light. The font in letter sets C and D was Calibri. The font in letter sets E and F was MS Gothic. The font in letter set F was hankaku (i.e., half-size letters) of katakana letters. The letter sets A–F were created by the authors in the present study based on the staircase stimuli.

**Table 2:**
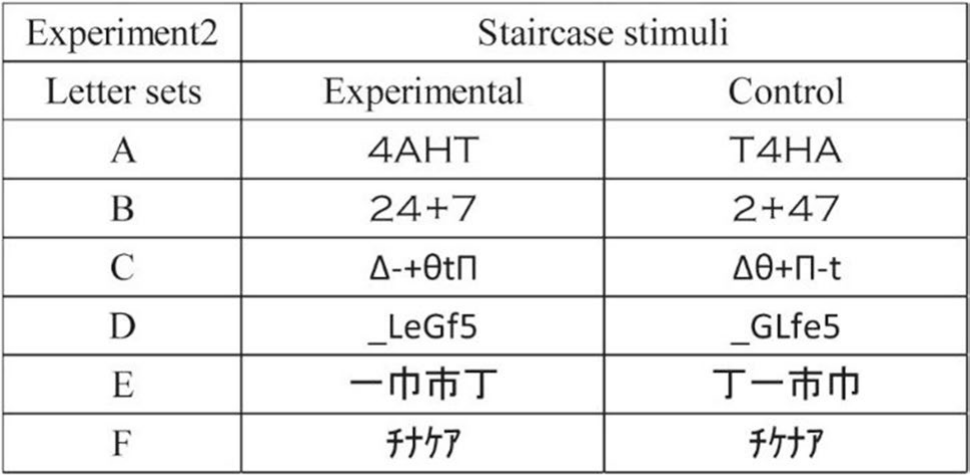
Stimuli of six letter sets used in Experiment 2

**Fig. 4:**
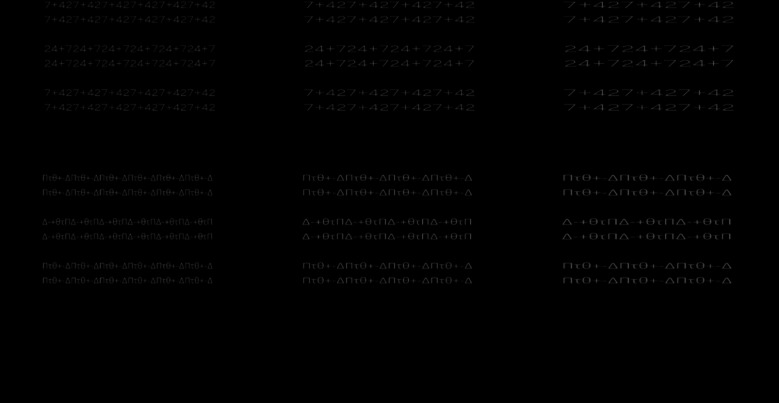
Stimuli in Experiment 2. Examples of the stimuli in the 1.0:1.0 (left), 1.5:1.0 (middle), and 2.0:1.0 (right) aspect ratios are shown. For example, the 2.0:1.0 ratio means the horizontal lengths in each letter were twice as long as those in the 1.0:1.0 ratio, whereas the horizontal length of each letter-row in the 2.0:1.0 ratio was same length to that in the 1.0:1.0 ratio. The vertical lengths of each letter of the 1.0:1.0 (left), 1.5:1.0 (middle), and 2.0:1.0 (right) ratios were physically the same. The letter-rows of letter set B “24+7” and letter set C “Δ-+θtΠ” were experimental and left conditions

There were five aspect ratios of each letter: 1.0:1.0, 1.5:1.0, 1.5:1.5, 2.0:1.0, and 2.0:2.0 H:V (horizontal:vertical) expansion ratios. To make stimuli of each aspect ratio condition, the displayed text of the 1.0:1.0 ratio condition was expanded in the horizontal and/or vertical dimension, and converted the images. Figure 4 shows the example of the stimuli of 1.0:1.0, 1.5:1.0, and 2.0:1.0 H:V ratios. The horizontal and vertical lengths of each letter were expanded or not expanded, and the number of repetitions of a letter set was same or different in order to make equal to total lengths of horizontal line segments in a letter-row of all ratio conditions in each letter set, and to present the display of the monitor.

The horizontal and vertical lengths of letters of letter set A in the 1.0:1.0 aspect ratio were equal to those in Experiment 1. Each observer completed two experimental sessions of 120 trials (3 letter sets × 2 standard stimuli × 5 ratios × 4 repetitions). The initial orientation of the line segment in each trial was the horizontal orientation. The line segment rotated 0.5 degrees clockwise and counterclockwise, respectively, by pressing the “4” and “6” keys on the numerical keypad. The order of the trials and the three letter sets in each session were randomized for each observer. The left or right conditions and upper or lower letter-rows location were counterbalanced. The horizontally and vertically averaged lengths of two letter-rows over six letter sets in the 1.0:1.0 aspect ratio were 12.5 and 1.7 degrees, respectively.

### Results and discussion

**Fig. 5:**
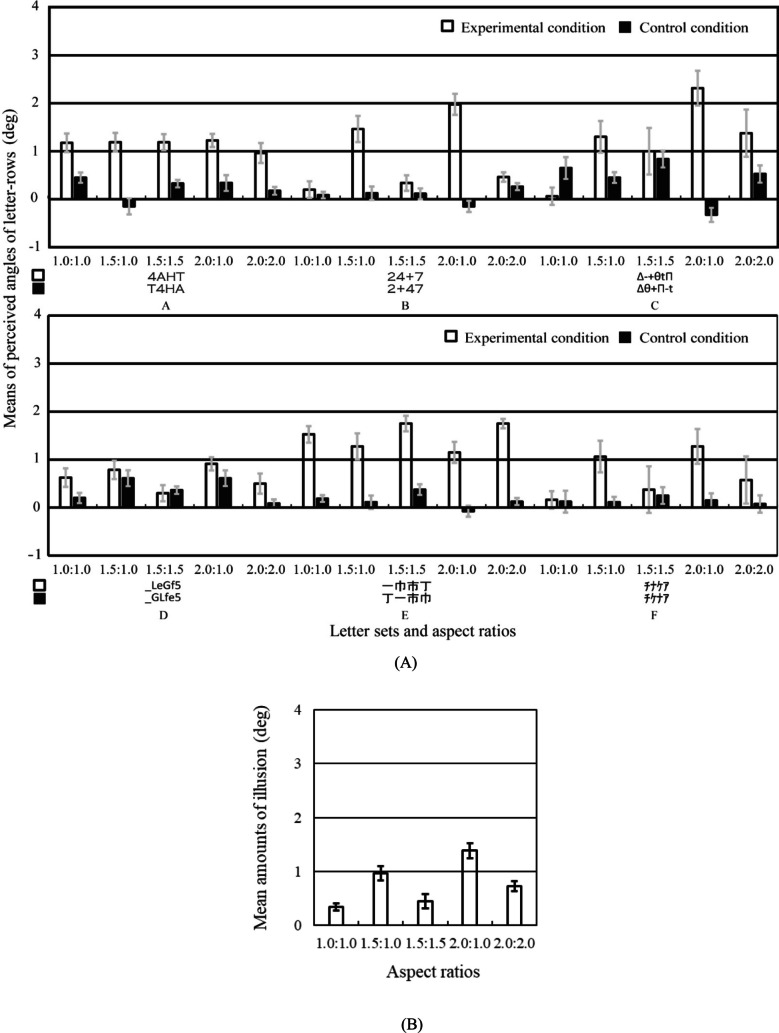
Results of Experiment 2. Error bars represent standard errors. (**A)** Means of the perceived angle of the central letter-rows averaged over the ten observers are plotted as a function of each letter set and aspect ratio condition separately for each standard stimulus. Positive and negative values indicate that the central two letter-rows were perceived to be tilted counterclockwise and clockwise, respectively. The 0 value indicates that the central two letter-rows were perceived to be not tilted and to be in the horizontal orientation. (**B**) Mean amounts of illusion averaged over the ten observers and six letter sets are plotted as a function of each aspect ratio condition. The amount of illusion was calculated as the subtraction of the perceived angle in the control condition from that in the experimental condition

Figure 5 shows the results of Experiment 2. A three-way repeated-measures ANOVA was performed on the perceived angle of the central letter-rows with standard stimulus (2 conditions), letter set (6 sets), and aspect ratio (5 ratios) as main factors.^1^ The main effect of standard stimulus was significant [*F*(1, 9) = 76.474,* p* <.001, *η*_p_^2^ =.90]. The interaction between standard stimulus, letter set, and aspect ratio was significant [*F*(20, 180) = 7.888, *p* <.001, *η*_p_^2^ =.47]. As shown in Fig. 5, the simple main effect tests of standard stimulus showed that the perceived angle was significantly larger in the experimental condition than that in the control condition in many letter set and ratio conditions (in letter sets A and E and in all ratios, in letter sets B and C and in 1.5:1.0 and 2.0:1.0 ratios, in letter set D and in 1.0:1.0, 2.0:1.0, and 2.0:2.0 ratios, and in letter set F and in 1.5:1.0, 2.0:1.0, and 2.0:2.0 ratios). The simple main effect test of standard stimulus showed that the perceived angle was significantly larger in the control condition than that in the experimental condition in 1.0:1.0 ratio and in letter set C condition. The results in letter sets A, C and D showed that the letter-row tilt illusion significantly occurred in the alphabetic letters of the Copperplate Gothic Light and Calibri fonts.

A one-way repeated-measures ANOVA was performed on the amount of illusion with aspect ratio (5 ratios) as a main factor. The amount of illusion was calculated as the subtraction of the perceived angle in the control condition from that in the experimental condition. The statistical units were the means averaged over six letter sets separately for each aspect ratio and each observer. The main effect of aspect ratio was significant [*F*(4*,* 36) = 24.443, *p* <.001, *η*_p_^2^ =.73]. As shown in Fig. 5, the results of multiple-comparison tests (Holm method) showed that the amount of illusion was significantly larger in the 2.0:1.0 aspect ratio than that in the 1.0:1.0, 1.5:1.0, 1.5:1.5, or 2.0:2.0 aspect ratio, in the 1.5: 1.0 aspect ratio than that in the 1.0:1.0, 1.5:1.5, or 2.0:2.0 aspect ratio, and in the 2.0:2.0 aspect ratio than that in the 1.0:1.0 aspect ratio.

The results showed that the amount of illusion increased as relative and absolute lengths of the horizontal line segments increased. The amount of illusion increased as relative length of the horizontal line segments in aspect ratios increased. The results showed that the amount of illusion was significantly larger in the 1.5:1.0 ratio than that in the 1.5:1.5 or 2.0:2.0 ratio and in the 2.0:1.0 ratio than that in the 2.0:2.0 ratio. The results indicated that even when the horizontal length was the same or shorter, the horizontal/vertical ratios of letters affected the illusion. On the other hand, the amount of illusion increased as absolute length of the horizontal line segments increased. The results showed that the amount of illusion was significantly larger in the 2.0:1.0 ratio than that in the 1.5:1.0 or 1.0:1.0 ratio, in the 1.5:1.0 ratio than that in the 1.0:1.0 ratio, and in the 2.0:2.0 ratio than that in the 1.0:1.0 ratio. In addition, the results also showed that sizes of letters significantly affected the illusion. The results showed that the amount of illusion was significantly larger in the 2.0:2.0 aspect ratio than that in the 1.0:1.0 aspect ratio.

Two one-way repeated-measures ANOVAs were performed on the amount of illusion with aspect ratio (3 ratios) as a main factor separately for each horizontal length and size. The main effects of aspect ratio in horizontal length (1.0:1.0, 1.5:1.0, 2.0:1.0 ratios) and size (1.0:1.0, 1.5:1.5, 2.0:2.0 ratios) were significant [*F*(2*,* 18) = 42.002, *p* <.001, *η*_p_^2^ =.82; *F*(2*,* 18) = 4.816, *p* =.002, *η*_p_^2^ =.35]. As shown in Fig. 5, the results of multiple-comparison tests (Holm method) in horizontal length showed that the amount of illusion was significantly larger in the 2.0:1.0 ratio than that in the 1.0:1.0 and 1.5:1.0 ratios and in the 1.5: 1.0 ratio than that in the 1.0:1.0 ratio. Moreover, the results of multiple-comparison tests (Holm method) in size showed that the amount of illusion was significantly larger in the 2.0:2.0 ratio than that in the 1.0:1.0 ratio. In the staircase stimuli, the amount of illusion was larger when horizontal lengths of each letter were longer, and the amount of illusion was larger when sizes of each letter were larger.

The reason for the results in 1.0:1.0 ratio and in letter set C condition was unclear. The results suggested an opposite illusion that the perceived angle was significantly larger in the control condition than that in the experimental condition.

These results in the staircase stimuli showed two main points: (1) The letter sets A, B, C, and D supported that the letter-row tilt illusion significantly occurred in alphabetic and numeric letters. (2) The amount of illusion increased as the relative and absolute lengths of the horizontal line segments increased.

## Experiment 3

**Fig. 6:**
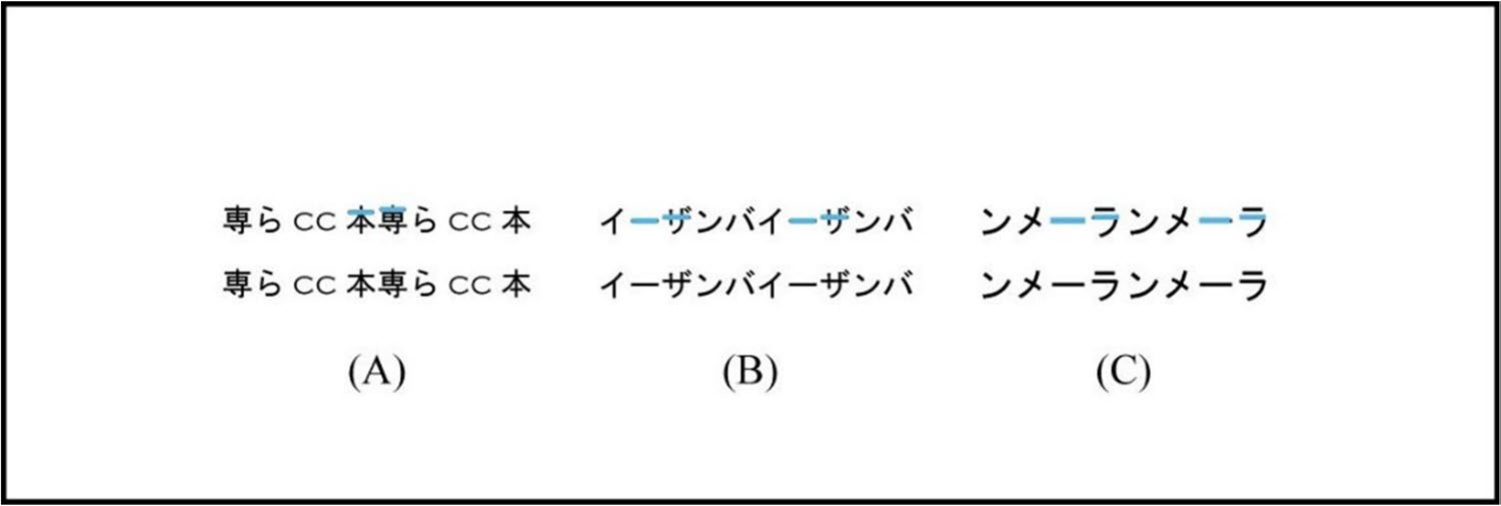
The stimuli with two sequential stairs. Light blue horizontal line segments indicate the two sequential stairs. The stimuli were used in the letter sets G, H, and I of non-staircase stimuli in Experiment 1, and were also used in the letter sets A, B, and C in Experiment 3

The purpose of Experiment 3 was to examine quantitatively the effects of the aspect ratios on the perceived angle of letter-rows in non-staircase stimuli in order to investigate whether there were different natures in the illusion between the staircase and non-staircase stimuli. The results in the staircase stimuli of Experiment 2 showed that the amount of illusion increased as the relative and absolute lengths of the horizontal line segments increased. Moreover, Experiment 3 also examined quantitatively whether only two sequential stairs or the other reasons in non-staircase stimuli generated the illusion in the letter sets G, H, and I of Experiment 1 that contained two sequential stairs in the non-staircase stimuli. Figure 6 shows the stimuli with two sequential stairs.

### Methods

The methods used in Experiment 3 were identical to those used in Experiment 2, except for the following points. Ten Japanese undergraduate students (18–22 years old, *M* = 20.0 years old, *SD* = 1.6; nine female, one male) participated as observers. The eight letter sets used in Experiment 3 were the non-staircase stimuli as shown in Table 3. In letter set A, the font of alphabetic letters was Copperplate Gothic Light, and the font of Chinese letters and a Japanese hiragana letter was MS Gothic as shown in Table 3. The font of letters in letter sets B, C, D, E, F, G, and H was MS Gothic.

**Table 3:**
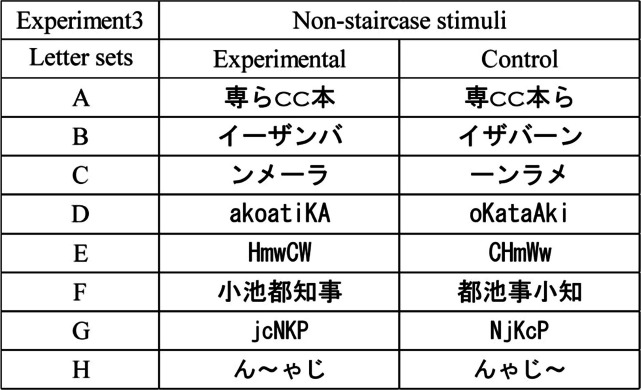
Stimuli of eight letter sets used in Experiment 3. The letter set G in the experimental condition was shown in Arai and Arai (2015). The letter sets D and H in the experimental condition were respectively shown on X (originally Twitter; flock pina [@flock_pina], 2018, 2020). The letter sets A, B, C, E, and F in the experimental condition were identical to letter sets G, H, I, K, and L in Experiment 1, respectively

**Fig. 7:**
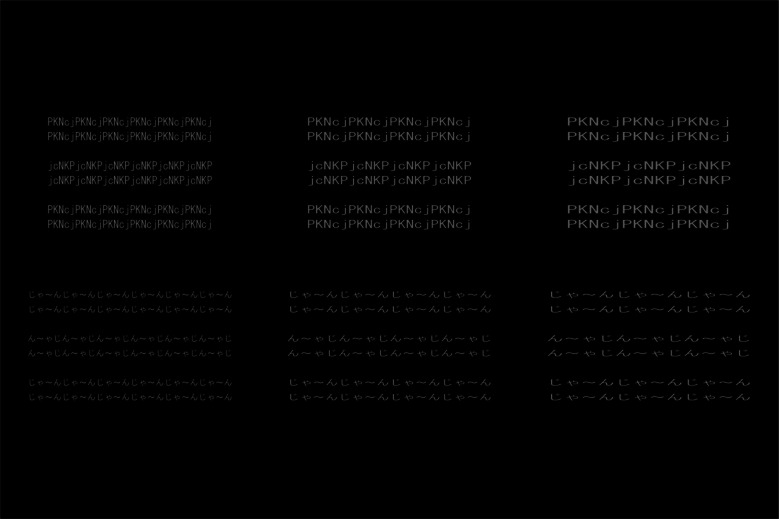
Stimuli in Experiment 3. Examples of the stimuli in the 1.0:1.0 (left), 1.5:1.0 (middle), and 2.0:1.0 (right) aspect ratios are shown. For example, the 2.0:1.0 ratio means the horizontal lengths in each letter were twice as long as those in the 1.0:1.0 ratio, whereas the horizontal length of each letter-row in the 2.0:1.0 ratio was same length to that in the 1.0:1.0 ratio. The vertical lengths of each letter of the 1.0:1.0 (left), 1.5:1.0 (middle), and 2.0:1.0 (right) ratios were physically the same. The letter-rows of the letter-sets G and H were experimental and left conditions

There were five aspect ratios of each letter: 1.0:1.0, 1.5:1.0, 1.5:1.5, 2.0:1.0, and 2.0:2.0 H:V expansion ratios. Figure 7 shows the example of the stimuli of 1.0:1.0, 1.5:1.0, and 2.0:1.0 H:V ratios. The horizontally and vertically averaged lengths of two letter-rows over eight letter sets in the 1.0:1.0 aspect ratio were 15.0 and 1.7 degrees, respectively. Each observer completed two experimental sessions of 160 trials (4 letter sets × 2 standard stimuli × 5 ratios × 4 repetitions). The order of the trials and the four letter sets in each session were randomized for each observer.

### Results and discussion

**Fig. 8:**
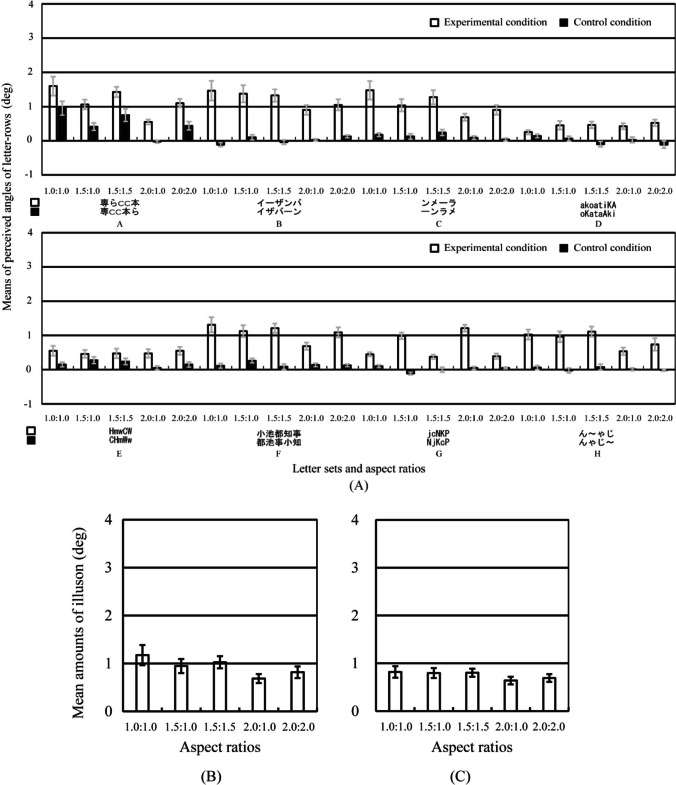
Results of Experiment 3. Error bars represent standard errors. (**A)** Means of the perceived angle of the central letter-rows averaged over the ten observers are plotted as a function of each letter set and aspect ratio condition separately for each standard stimulus. Letters on the x-axis A, B, C, D, E, F, G, and H correspond to those of the letter sets in Table 3. Positive and negative values indicate that the central two letter-rows were perceived to be tilted counterclockwise and clockwise, respectively. The 0 value indicates that the central two letter-rows were perceived to be not tilted and to be in the horizontal orientation. (**B)** Mean amounts of illusion averaged over the ten observers and eight letter sets are plotted as a function of each aspect ratio condition. The amount of illusion was calculated as the subtraction of the perceived angle in the control condition from that in the experimental condition. **(C**) Mean amounts of illusion averaged over the ten observers and three letter sets A, B, and C are plotted as a function of each aspect ratio condition

Figure 8 shows the results of Experiment 3. A three-way repeated-measures ANOVA was performed on the perceived angle of the central letter-rows with standard stimulus (2 conditions), letter set (8 sets), and aspect ratio (5 ratios) as main factors.^2^ The main effect of standard stimulus was significant [*F*(1, 9) = 70.538,* p* <.001, *η*_p_^2^ =.89]. The interaction between standard stimulus, letter set, and aspect ratio was significant [*F*(28, 252) = 6.437, *p* <.001, *η*_p_^2^ =.42]. As shown in Fig. 8, the simple main effect tests of the standard stimulus showed that the perceived angle was significantly larger in the experimental condition than that in the control condition in many letter set and ratio conditions (in letter sets A, B, C, F, G, and H and in all ratios, in letter set D and in 1.5:1.0, 1.5:1.5, 2.0:1.0, and 2.0:2.0 ratios, and in letter set E and in 1.0:1.0, 2.0:1.0, and 2.0:2.0 ratios). The results in the letter sets D, E, and G showed that the illusion occurred significantly in the alphabetic letters and non-staircase stimuli. The results in the letter set E supported that the font of letters affected the illusion (Arai & Arai, 2018).

A one-way repeated-measures ANOVA was performed on the amount of illusion with aspect ratio (5 ratios) as a main factor. The amount of illusion was calculated as the subtraction of the perceived angle in the control condition from that in the experimental condition. The statistical units were the means averaged over the eight letter sets separately for each aspect ratio and each observer. The main effect of aspect ratio was significant [*F*(4*,* 36) = 4.559, *p* =.004, *η*_p_^2^ =.34]. The results of multiple-comparison tests (Holm method) showed that the amount of illusion was significantly larger in the 1.0:1.0, 1.5:1.0, and 1.5:1.5 ratios than that in the 2.0:1.0 ratio. The results showed that the amount of illusion in the non-staircase stimuli had different tendencies in aspect ratios from that in the staircase stimuli in Experiment 2.

In the letter sets A, B, and C with the two sequential stairs, a one-way repeated-measures ANOVA was performed on the amount of illusion with aspect ratio (5 ratios) as a main factor, in order to examine whether only two sequential stairs or the other factors in the non-staircase stimuli generated the illusion. The statistical units were the means averaged over the letter sets A, B, and C separately for each aspect ratio and each observer. The main effect of aspect ratio was significant [*F*(4*,* 36) = 5.230, *p* =.002, *η*_p_^2^ =.37]. The results of multiple-comparison tests (Holm method) showed that the amount of illusion was significantly larger in the 1.0:1.0 and 1.5:1.5 ratios than that in the 2.0:1.0 ratio. The results in the letter sets A, B, and C showed that the amount of illusion had different tendencies in the aspect ratios from that in the staircase stimuli in Experiment 2. The results suggested that the only two sequential stairs did not generate the illusion in the letter sets A, B, and C and the other factors generated the illusion in the letter sets A, B, and C.

Two one-way repeated-measures ANOVAs were performed on the amount of illusion with aspect ratio (3 ratios) as a main factor separately for each horizontal length and size. The main effect of aspect ratio in horizontal length (1.0:1.0, 1.5:1.0, 2.0:1.0) or size (1.0:1.0, 1.5:1.5, 2.0:2.0) was or was not significant [*F*(2*,* 18) = 7.471, *p* =.004, *η*_p_^2^ =.45; *F*(2*,* 18) = 2.970, *p* =.08, *η*_p_^2^ =.25]. As shown in Fig. 8, the results of multiple-comparison tests (Holm method) in horizontal ratio showed that the amount of illusion was significantly larger in the 1.0:1.0 and 1.5:1.0 ratios than that in the 2.0:1.0 ratio. In the non-staircase stimuli, the amount of illusion was larger when horizontal lengths of each letter were shorter, and sizes of each letter did not significantly affect the amount of illusion.

The amount of illusion in the non-staircase stimuli had different tendencies in aspect ratios from that in the staircase stimuli in Experiment 2. In Experiment 2, the results showed that the amount of illusion in staircase stimuli increased as the relative and absolute lengths of the horizontal line segments increased. On the other hand, in Experiment 3, the results showed that the amount of illusion in the non-staircase stimuli was significantly larger in the 1.0:1.0, 1.5:1.0, and 1.5:1.5 ratios than that in the 2.0:1.0 ratio. These results suggested that the illusion in non-staircase stimuli would occur for different reasons from that in staircase stimuli.

These results in the non-staircase stimuli showed three main points: (1) In the letter sets D, E, and G, the illusion significantly occurred in alphabetic letters and non-staircase stimuli. The amount of illusion in the non-staircase stimuli was significantly larger in the 1.0:1.0, 1.5:1.0, and 1.5:1.5 aspect ratios than that in the 2.0:1.0 aspect ratio. (3) The amount of illusion in the non-staircase stimuli had different tendencies in aspect ratios from that in the staircase stimuli in Experiment 2.

These results suggest that the illusion in non-staircase stimuli would occur for different reasons from that in staircase stimuli.

## General discussion

The present study showed that aspect ratios of letters significantly affected the letter-row tilt illusion. In Experiment 1, the results quantitatively showed that the illusion significantly occurred in each five of six letter sets of staircase and non-staircase stimuli. In Experiment 2, the results showed that the amount of illusion in staircase stimuli increased as the relative and absolute lengths of the horizontal line segments increased. In Experiment 3, the results showed that the amount of illusion in the non-staircase stimuli had different tendencies in aspect ratios from that in the staircase stimuli in Experiment 2.

The results in the present study quantitatively supported the previous study and description regarding the following four points. (1) The illusion occurred in the non-staircase stimuli (e.g., Arai & Arai, 2015). In Experiments 1 and 3, the results in the non-staircase stimuli of all nine letter sets showed that the perceived angle was significantly larger in the experimental condition than that in the control condition. (2) The amount of illusion increased as the absolute length of the horizontal line segments increased (Kohara, 2006). In Experiment 2, the results in the staircase stimuli showed that the amount of illusion increased as the relative and absolute lengths of the horizontal line segments increased.^3^ (3) The font of letters affected the illusion (Arai & Arai, 2018). The results showed that the illusion in the letter sets “HmwCW” did not significantly occur in Copperplate Gothic Light font in Experiment 1, and significantly occurred in MS Gothic font in Experiment 3. (4) The illusion occurred in alphabetic and numeric letters (Arai & Arai, 2015).

The present study supported the staircase hypothesis in the illusion with the staircase stimuli. In Experiment 2, the results in the staircase stimuli showed that the amount of illusion increased as the relative and absolute lengths of the horizontal line segments increased. Especially, the finding that the amount of illusion increased as the relative length of the horizontal line segments increased confirmed the critical role of horizontal segments on the illusion in the staircase stimuli. Moreover, the results of Experiment 2 showed that the staircase stimuli generated the illusion in many letter set and ratio conditions, but the staircase stimuli did not generate the illusion in any conditions. In Experiment 3, the results in the letter sets with two sequential stairs suggested that factors other than the two sequential stairs generated the illusion. These results also suggested that the presence of staircase substructures in the stimuli was not always particularly visually salient and it tended to be obscured by the presence of many other line segments of letters and geometrical substructures of letter-rows.

The main finding of the present study was that there were different natures between the illusion in staircase and non-staircase stimuli. In Experiment 1, the results showed that the amount of illusion in the staircase stimuli was significantly larger than that in the non-staircase stimuli. In Experiment 2, the results in the staircase stimuli showed that the amount of illusion increased as the relative and absolute lengths of the horizontal line segments increased. On the other hand, in Experiment 3, the results in non-staircase stimuli showed that the amount of illusion was significantly larger in the 1.0:1.0, 1.5:1.0, and 1.5:1.5 ratios than that in the 2.0:1.0 ratio. The results showed that the amounts of illusion had different tendencies in aspect ratios in staircase and non-staircase stimuli. Moreover, the results in the letter sets with two sequential stairs suggested that the two sequential stairs did not generate the illusion. These results suggested that the mechanism underlying the illusion differed in staircase and non-staircase stimuli. In addition, these results also suggested that the staircase hypothesis did not account for the generation of the illusion in non-staircase stimuli.

There is a possible explanation for the natures in aspect ratios of the illusion in staircase and non-staircase stimuli, i.e., ensemble processing for orientation. However, ensemble processing for orientation would not generate the different natures in aspect ratios between the illusions in staircase and non-staircase stimuli, since the same letters were used in the experimental and control conditions in the present study. In addition, Allik et al. (2023) reported that there was no evidence of arithmetic averaging in ensemble processing for orientation.

On the other hand, the results of the present study also showed that there was a common nature of the illusion in staircase and non-staircase stimuli. The results in staircase and non-staircase stimuli suggested that the illusion was attributed to the order of letters (i.e., global structure of letter-rows) rather than the aggregate of the effect of each letter, since the difference in the stimuli between the experimental and control conditions was the order of letters. The global structure of letter-rows was self-evident in the staircase stimuli with sequential stairs, whereas it was not self-evident in the non-staircase stimuli. The non-staircase stimuli also contained global structures that could not be seen, and generated the illusion.

The present study showed that aspect ratios of letters significantly affected the letter-row tilt illusion, and suggested that the mechanism underlying the letter-row tilt illusion with and without the staircase structure had different and common processes. Both in the illusion with and without a staircase structure, global structures of letter-rows generated the illusion, whereas the illusion with and without a staircase structure would be attributed to the different types of global structures. Further study is necessary to examine the different and common natures in the illusion with and without a staircase structure in order to clarify the mechanism underlying the letter-row tilt illusion and visual processing for orientation in human perception.^4^

## Data Availability

None of the data for the experiments in the present study is available.

## Footnotes

1 The main effects of letter set and aspect ratio were significant [*F*(5, 45) = 3.819,* p* =.006, *η*_p_^2^ =.30; *F*(4, 36) = 9.159,* p* <.001, *η*_p_^2^ =.50]. The interactions between standard stimulus and letter set, between standard stimulus and aspect ratio, and between letter set and aspect ratio were significant [*F*(5, 45) = 7.254, *p* <.001, *η*_p_^2^ =.45; *F*(4, 36) =24.443, *p* <.001, *η*_p_^2^ =.73; *F*(20, 180) = 6.636, *p* <.001, *η*_p_^2^ =.42]. In the six letter sets A–F, the simple main effect tests of standard stimulus showed that the perceived angle was significantly larger in the experimental condition than that in the control condition [*F*(1*,* 54) = 45.724, 64.538, 10.186, 7.937, 63.886, 19.720; *p* <.001, <.001, =.011, =.020, <.001,.002]. In the five aspect ratios, the simple main effect tests of standard stimulus showed that the perceived angle was significantly larger in the experimental condition than that in the control condition [*F*(1*,* 45) = 30.548, 51.124, 12.170, 93.578, 59.789; *p* <.001, <.001, =.007, <.001, <.001].

2 The main effects of letter set and aspect ratio were significant [*F*(7, 63) =15.837, *p <*.001, *η*_p_^2^ =.64; *F*(4, 36) =11.849,* p* <.001, *η*_p_^2^ =.57]. The interactions between standard stimulus and letter set, between standard stimulus and aspect ratio, and between letter set and aspect ratio were significant [*F*(7, 63) = 9.774, *p* <.001, *η*_p_^2^ =.52; *F*(4, 36) = 4.559, *p =*.004, *η*_p_^2^ =.34; *F*(28, 252) = 7.909, *p* <.001, *η*_p_^2^ =.47]. In the eight letter sets A–H, the simple main effect tests of standard stimulus showed that the perceived angle was significantly larger in the experimental condition than that in the control condition [*F*(1*,* 72) = 80.319, 40.950, 32.042, 23.228, 12.160, 38.978, 135.375, 53.261; *p* <.001, <.001, <.001, <.001, =.007, <.001, <.001, <.001]. In the five aspect ratios, the simple main effect tests of standard stimulus showed that the perceived angle was significantly larger in the experimental condition than that in the control condition [*F*(1*,* 45) = 29.874, 17.009, 58.983, 33.363, 110.494; *p* <.001, =.003, <.001, <.001, <.001].

3 Kohara (2006) suggested the similarity between the letter-row tilt illusion and the Popple illusion or phase-shift illusion (Popple & Levi, 2000). That is, the amounts of Popple illusion increased and decreased when there were more Gabor patches and the distances between the patches were further, respectively (Popple & Levi, 2000). In Experiment 2 of the present study, the results in the staircase stimuli showed that the amount of the letter-row tilt illusion increased as the relative and absolute lengths of the horizontal line segments increased. The results in absolute length in the letter-row tilt illusion might be attributed to common processes to the Popple illusion, whereas it was unclear whether the results in relative length were attributed to common processes to the Popple illusion. Moreover, the results in non-staircase stimuli would be attributed to different processes to the Popple illusion.

4 In addition, there are many tilt illusions (e.g., the Popple illusion, Zöllner illusion, Café Wall illusion, and twisted cord illusion). The stimuli of the letter-rows tilt illusion were mainly letters that are more complex than the stimuli of the other tilt illusion. Kohara (2006) suggested the similarity between the letter-row tilt illusion with the staircase structure and the Popple illusion or phase-shift illusion (Popple & Levi, 2000). In the other tilt illusions, more elaborate experiments were needed to examine the similarity between the letter-row tilt illusion and the other tilt illusions.
